# Paroxysmal Sneezing at the Onset of Syncopes and Transient Ischemic Attack Revealing a Papillary Cardiac Fibroelastoma

**DOI:** 10.1155/2014/734849

**Published:** 2014-06-17

**Authors:** Stéphane Mathis, Matthias Lamy, Jonathan Ciron, Anna Iljicsov, Roxana Arjmand, Pierre Agius, Jean-Philippe Neau

**Affiliations:** Department of Neurology, University of Poitiers, CHU Poitiers, 2 Rue de la Milétrie, 86021 Poitiers Cedex 05, France

## Abstract

Sneezing can at times be associated with neurological disorders. The “sneeze center” is localized in the lateral medulla. We report the case of a 50-year-old man who presented three episodes of sneezing, two of them followed by an episode of transient gait instability and dizziness and the third one followed by an episode of transient left hemiparesis due to fibroelastoma of the aortic cardiac valve. To the best of our knowledge, this is the first description of a transient ischemic attack due to cardiac papillary fibroelastoma and revealed by violent episodes of sneezing.

## 1. Introduction

Sneezing is a protective physiological respiratory reflex that consists of a sequence of mouth closing, a single slow deep inspiration or series of inspiratory efforts, and then an explosive expiratory effort with closed glottis [1]; the consequence is increased intrapulmonary pressure [2]. However, this phenomenon can be modulated by voluntary cortical activity [3]. In humans, its topography remains uncertain, but cases of “inability to sneeze” have been observed after an inflammatory lesion of the rostral dorsolateral medulla [4] or an ischemic lesion of the lateral medulla [5]. Subsequent to this first step, an efferent (or respiratory) phase occurs with recruitment of a number of inspiratory and expiratory neurons [2]. Here we describe transient neurological symptoms following sneezing and finally revealing a cardiac papillary fibroelastoma.

## 2. Case Report

A 50-year-old man presented with three stereotyped episodes of transient neurological disorders preceded by sneezing. The first one occurred in the morning; it began with a sudden diffuse warm sensation accompanied by sweating and was immediately followed by four sneezes, gait instability, and dizziness. All of these neurological symptoms totally disappeared in less than 30 minutes. A second and similar transient episode (30 minutes) occurred a few days later in the morning, immediately after sneezing. Three weeks later, a third episode was observed (once again after sneezing), but with transient gait instability and left hemiparesis. After one hour, all of these signs had totally disappeared. Between each of the three transitory episodes, the neurological examination was normal.

The patient's medical history was unremarkable except for treated arterial hypertension. In his family's medical background, there had been cases of arterial hypertension (mother and sister), cerebral aneurysm (mother), and multiple sclerosis (father).

Electrocardiographic monitoring was normal. Carotid and vertebral artery ultrasonography showed moderate atheroma without stenosis. Magnetic resonance imaging (MRI) of the brain was normal and angiographic sequences showed no vascular abnormality in the cervical or cerebral arteries. Transesophageal echocardiographic examination revealed a homogeneous and slightly mobile nodular mass (diameter = 6 millimeters) attached to the ventral part of the right anterior cusp of the aortic valve, without hindering the valvular function. The interauricular septum was normal, without patent foramen ovale. There was no atheroma of the aortic arch.

In this case, which involved a valvular intracardiac tumor with potentially serious embolic complications, surgery was decided upon. Resection of a voluminous tumor of the right coronary cusp of the aortic valve was carried out; a smaller tumor of the noncoronary cusp of the aortic valve was likewise removed. Pathological study disclosed a papillary fibroelastoma of the coronary cusp of the aortic valve. The tumor of the noncoronary cusp consisted in fibrous tissue alone. Two years after cardiac surgery, no further transient neurological episodes had been observed, even after sneezing.

## 3. Discussion

In our patient, the first two neurological episodes probably corresponded to syncope, but the third was more likely an episode of transient vertebrobasilar ischemia. However, even though the patient presented with a potentially embolic intracardiac tumor, with a strictly normal brain MRI, there was no argument for a stroke.

Papillary fibroelastoma is a rare intracardiac tumor (usually presenting as a mobile mass) and most often observed in patients over 50 years old [6]. It is the second most common primary cardiac tumor and the most common tumor involving the cardiac valves; it represents about 10% of all cardiac primary neoplasms [7]. It originates in the valve endothelium and is usually found on the valve leaflets (involving mostly the aortic and mitral valves) [6] and less often on the chordae tendineae and in the ventricles [7]. Most fibroelastomas are benign and asymptomatic (usually found incidentally by echocardiography, with an incidence of 0.019%) [8]. That said, at times, depending on the location and/or dimensions of the tumor, their presence can be signalled by syncope [9–11] and, more rarely, by distal embolisation of the tumor causing transient ischemic attack, stroke, myocardial infarction, or sudden death [7, 8, 12, 13]. For these reasons, whenever such an intracardiac tumor is diagnosed, surgical treatment must be considered, even in asymptomatic cases: the operation is usually a simple excision [14], at times with leaflet repair [12].

Like coughing, sneezing is a well-recognized but hardly common cause of syncope; the previously suggested mechanism of a neurally mediated situational syncope is transient venous obstruction due to increased intrathoracic pressure, which may contribute to cerebral hypoperfusion [15]. In fact, sneezing, as in the Valsalva manoeuvre, is associated with increased intrathoracic pressure, arterial pressure rising to 80–90 mmHg [1], and venous pressure as well. Furthermore, the rise in intrathoracic pressure is associated with a rise in intracranial pressure [1]. Moreover, it has been confirmed that coughing diminishes phasic carotid blood velocity and reduces cerebral perfusion [16]. In the past, “inability to sneeze” was observed after cerebral lesions [4, 5] and a variety of neurological disorders (other than syncope) were reported after sneezing; they included vertigo and deafness, subarachnoid haemorrhage and headache, drop attacks (in cases of Arnold-Chiari malformation), cerebrospinal fluid rhinorrhoea and pneumocephalus, or sudden quadriplegia after acute cervical disc herniation [1, 17]. Cardiovascular complications were likewise reported after sneezing; they included coronary artery spasms, acute aortic dissection [18], and cerebral ischaemic stroke [19]. For example, Gutowski et al. observed a left-sided upper cervical cord lesion probably due to partial left vertebral artery dissection after sneezing in a 35-year-old man [20], and Harrison reported a carotid stroke after sneezing revealing a carotid artery stenosis [1]. Another case was observed in a context of intracranial aneurysms [1]. Moreover, central retinal artery occlusion due to a calcific embolus in a case of calcific aortic valve disease was described immediately after 2 violent sneezes [1]. While salves of sneezing may provoke neurological disorders, they may also represent the initial manifestations of a vertebrobasilar ischemic attack, rather than their consequence; in that case, sneezing may be due to irritation of the lateral medulla corresponding to the “sneezing center” [5].

To sum up, our patient presented two possible causes of syncope: papillary cardiac fibroelastoma and violent episodes of sneezing. In the final analysis, the syncopes and the subsequent transient ischemic attack were in all likelihood directly due to the papillary fibroelastoma but were revealed only by the episodes of violent sneezing (because of the hemodynamic modifications induced by the Valsalva manoeuvre). To our knowledge, there has been no published report on this type of clinical event, which leads us to conclude that sneezing may not always be as benign as it seems.
